# Supplementary material for: Deletion of 3p13-14 locus spanning FOXP1 to SHQ1 cooperates with PTEN loss in prostate oncogenesis
Source: Nat Commun. 2017 Oct 20;8:1081. doi: 10.1038/s41467-017-01198-9 (PMC5651901; doi:10.1038/s41467-017-01198-9)
Supplement: Supplementary file 1 — Supplementary information [file 41467_2017_1198_MOESM1_ESM.pdf]

Supplementary Figures

Supplementary Figure 1. Conditional deletion of the syntenic 3p13-14 genomic locus spanning *SHQ1* to *FOXP1* in transgenic mice. Diagram of the syntenic genomic locus from *FOXP1* to *SHQ1* in (**a**) *H. sapiens* and (**b**) *M. musculus*. RefSeq genes at this locus, *FOXP1, EIF4E3, GPR27, PROK2, RYBP*, and *SHQ1*, are shown. (**c**) Targeting diagram of *SHQ1* locus. (**d**) Diagram of the transgenic locus in *Foxp1-Shq1^{flox}* mice. Red triangles indicate loxP sites. The transgenic unrecombined and recombined loci observed by PCR in mouse prostate upon Pb-Cre introduction are shown. (**e**) Deletion of region from *Shq1* through *Foxp1* outer loxP sites (top), *Foxp1* alone (middle), and *Shq1* alone (bottom), shown by PCR of genomic DNA from transgenic mouse prostates.

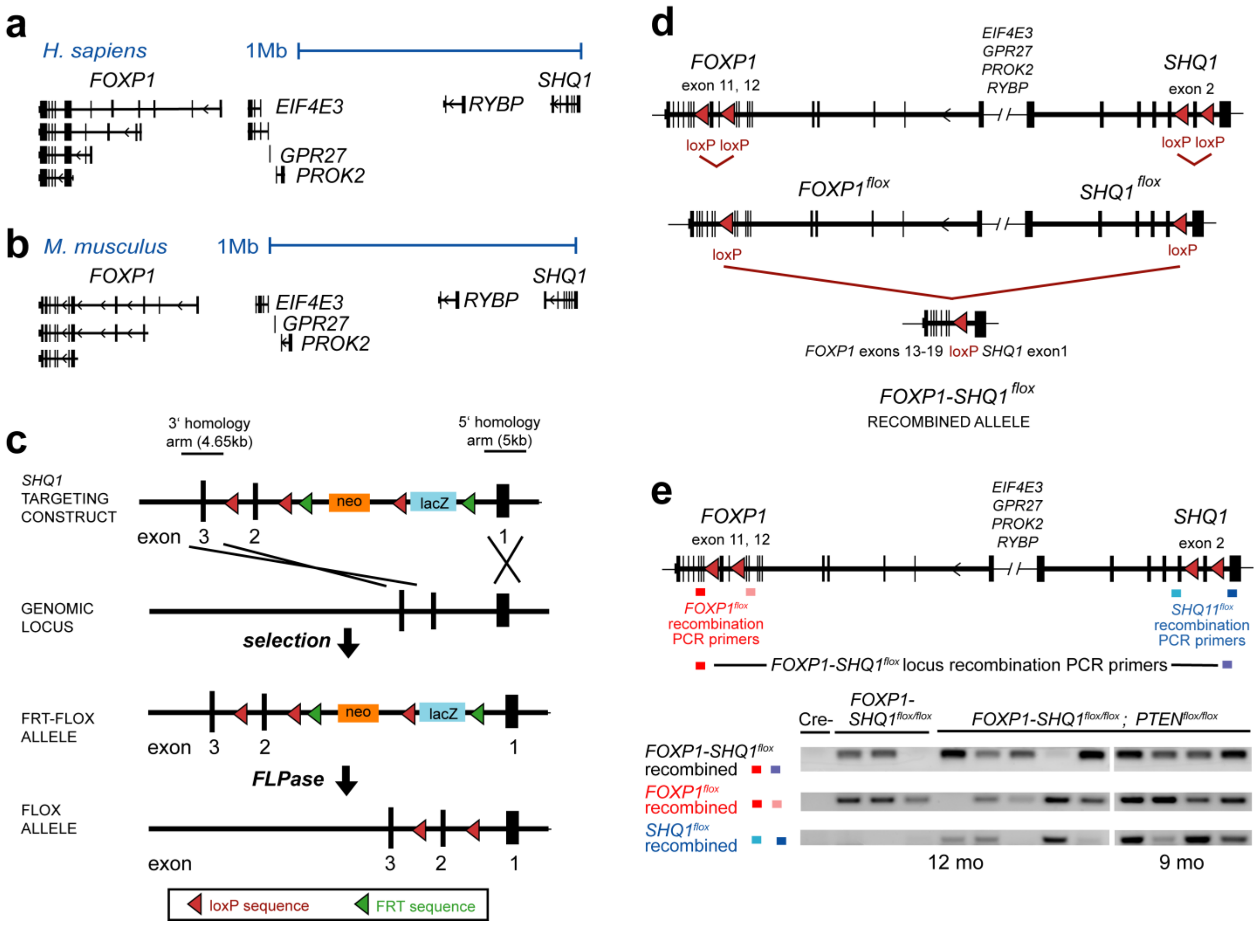

Supplementary Figure 2. Combined *Pten* and *Foxp1-Shq1* locus loss accelerates development of murine prostate carcinoma compared to *Pten* loss alone. Plots of the cumulative incidence of freedom from disease show the progression to (**a**) intraductal carcinoma and (**b**) invasive carcinoma in *Pten*$^{flox/flox}$ (blue; total n= 23; 6 mo, n = 7; 9 mo, n=8; 12 mo, n=8) and *Foxp1-Shq1*$^{flox/flox}$;*Pten*$^{flox/flox}$ (red; total n= 28; 6 mo, n = 8; 9 mo, n=10; 12 mo, n=10) mouse prostates at 6, 9, and 12 months. Log rank (Mantel Cox) P-value shown.

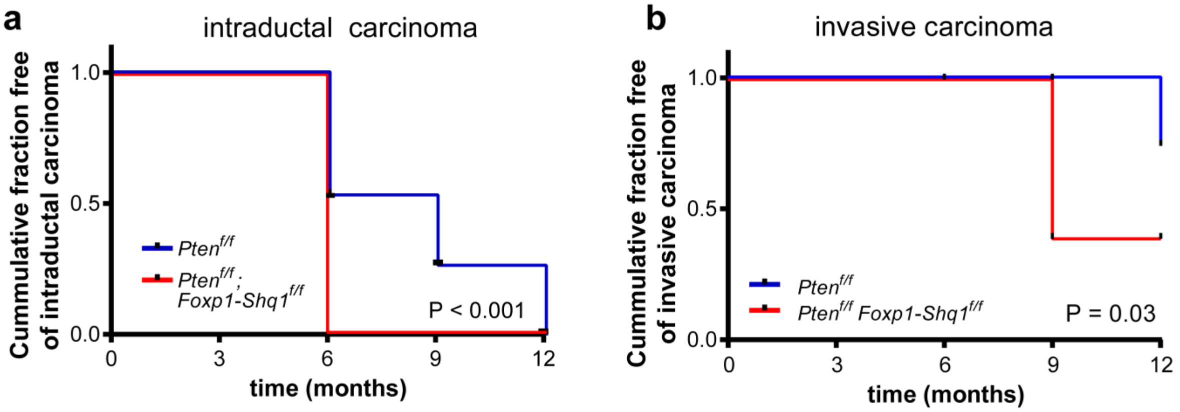

Supplementary Figure 3. Proliferation is increased in *Foxp1-Shq1*$^{flox/flox}$*;Pten*$^{flox/flox}$ murine prostate tumors compared to *Pten*$^{flox/flox}$ tumors. Quantification of Ki67-positivity (% Ki67-positive tumor nuclei) in wildtype, *Foxp1-Shq1*$^{flox/flox}$, *Pten*$^{flox/flox}$, and *Foxp1-Shq1*$^{flox/flox}$*;Pten*$^{flox/flox}$ mice was carried out by Ki67 immunohistochemistry on slides of tumor from mice aged 9 and 12 months. t-test, *, P<0.05. **, P<0.01.

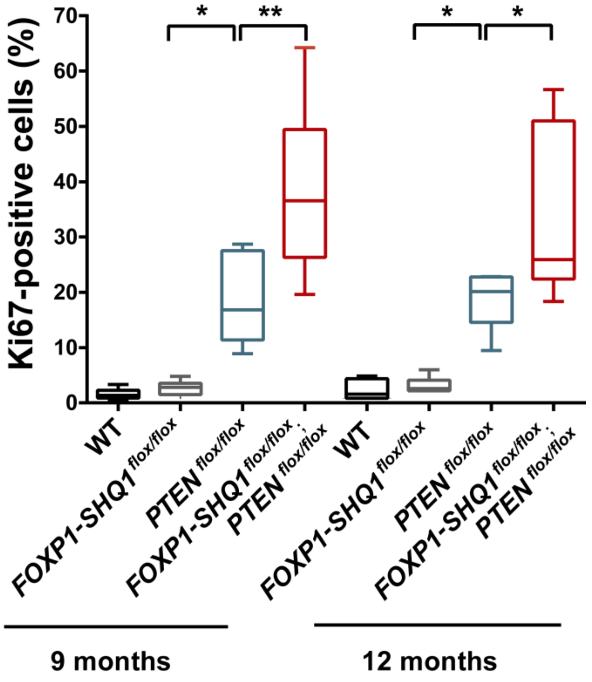

Supplementary Figure 4. Luminal marker expression is retained in murine prostate tumors that have *Foxp1-Shq1* and *Pten* deleted. CK 8/18 (luminal) and keratin 5 and 14 (basal) expression are shown by IHC along with H&E staining in wildtype, *Foxp1-Shq1*$^{flox/flox}$, *Pten*$^{flox/flox}$, and *Foxp1-Shq1*$^{flox/flox}$;*Pten*$^{flox/flox}$ mouse prostate at 12 months. The first column for each genotype corresponds to the same region shown in other figures showing IHC. Scale bars, 50 µm. Insets, 4X magnification of panel.

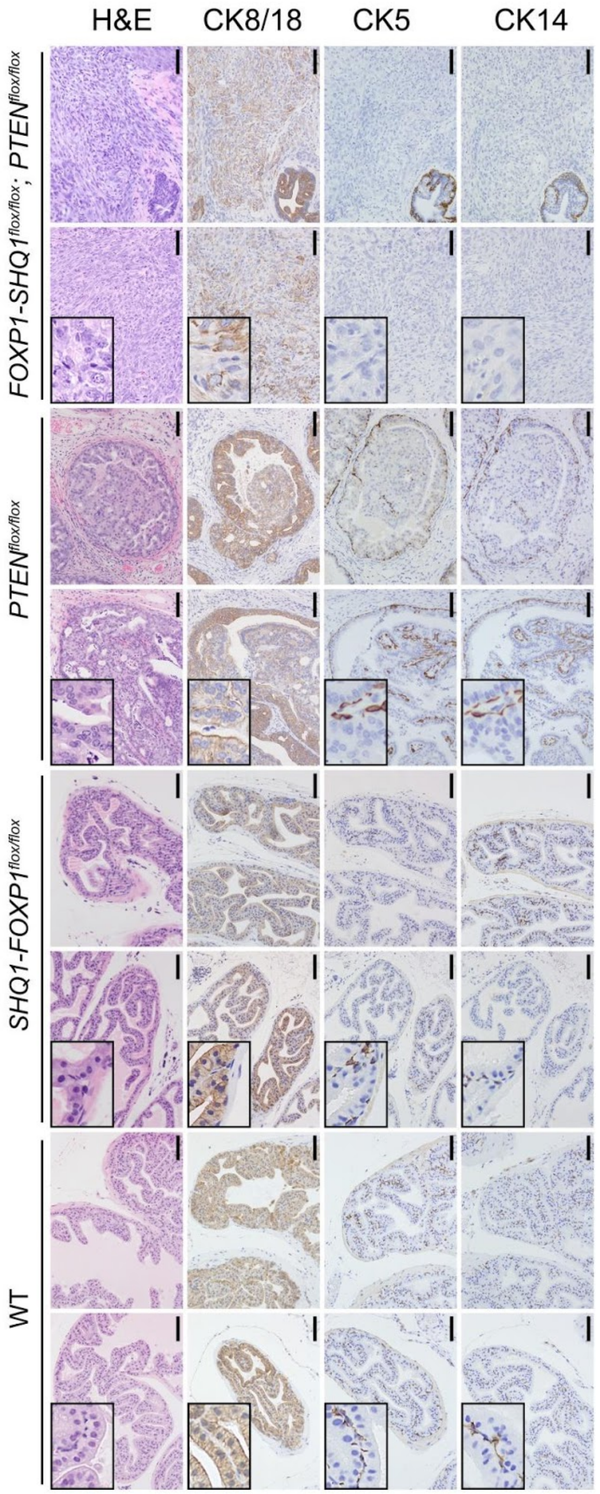

Supplementary Figure 5. Gene expression changes in *Foxp1-Shq1*$^{flox/flox}$;*Pten*$^{flox/flox}$ murine tumors compared to *Pten*$^{flox/flox}$ tumors. (**a**) Unsupervised hierarchical clustering of RNA-seq gene expression in *Foxp1-Shq1*$^{flox/flox}$;*Pten*$^{flox/flox}$ and *Pten*$^{flox/flox}$ mouse prostates at 12 months. The thousand most variant genes by RNA-seq shown. (**b**) FOXP3 target genes are downregulated in *Foxp1-Shq1*$^{flox/flox}$;*Pten*$^{flox/flox}$ murine tumors compared to *Pten*$^{flox/flox}$ tumors. The signature of FOXP3 bound genes (ZHENG_BOUND_BY_FOXP3, genes whose promoters are bound by FOXP3 based on ChIP-chip analysis in murine CD4+ T-cells[1]) is enriched in prostate tumors from mice *Pten*$^{flox/flox}$ compared to those of *Foxp1-Shq1*$^{flox/flox}$;*Pten*$^{flox/flox}$ mice at 12 months, as assessed by GSEA after RNA-seq (P <0.001, FDR = 0.003). (**c**) De-differentiation related gene expression signatures are enriched in *Foxp1-Shq1*$^{flox/flox}$;*Pten*$^{flox/flox}$ murine tumors compared to *Pten*$^{flox/flox}$ tumors, as assessed by GSEA after RNA-seq. Mammary stem cell gene expression (left, PECE_MAMMARY_STEM_CELL_UP, genes consistently up-regulated in normal mammary stem cells from cultured mammospheres[2]) is enriched in prostate tumors from *Foxp1-Shq1*$^{flox/flox}$;*Pten*$^{flox/flox}$ mice compared to *Pten*$^{flox/flox}$ mice at 12 months (P <0.001, FDR <0.001). Expression of genes repressed by differentiation-inducing drug ATRA (right, MARTENS_TRETINOIN_RESPONSE_DN, genes down-regulated in acute promyelocytic leukemia NB4 cells in response to tretinoin[2]) enriched in prostate tumors from *Foxp1-Shq1*$^{flox/flox}$;*Pten*$^{flox/flox}$ mice compared to *Pten*$^{flox/flox}$ mice at 12 months (P <0.001, FDR <0.001). (**d**) mTORC1 induced gene expression, defined by everolimus and rapamycin treatment is increased in *Foxp1-Shq1*$^{flox/flox}$;*Pten*$^{flox/flox}$ tumors mice relative to *Pten*$^{flox/flox}$ tumors. mTORC1 inhibition signature of everolimus treatment (MTOR_UP.V1_UP, genes up-regulated by everolimus in mouse prostate[3]) is enriched in tumors of *Pten*$^{flox/flox}$ mice compared to those of *Foxp1-Shq1*$^{flox/flox}$, *Pten*$^{flox/flox}$ mice at 12 months, as assessed by GSEA after RNA-seq (left; P <0.001, FDR = 0.019). mTORC1-induced gene expression repressed by rapamycin (PENG_RAPAMYCIN_RESPONSE_DN, genes down-regulated in B-lymphoma BJUB cells in response to rapamycin) is enriched in prostate tumors from *Foxp1-Shq1*$^{flox/flox}$;*Pten*$^{flox/flox}$ mice relative to *Pten*$^{flox/flox}$ mice at 12 months (right; P <0.001, FDR <0.001). (**e**) Genes repressed by MEK activation (MEK_UP.V1_DN, genes down-regulated in MCF-7 breast cancer cells upon over-expression of constitutively active MAP2K1[4]) are under-enriched (i.e., also repressed) in prostate tumors from *Foxp1-Shq1*$^{flox/flox}$;*Pten*$^{flox/flox}$ mice relative to *Pten*$^{flox/flox}$ mice at 12 months, as assessed by GSEA after RNA-seq (P = 0.004, FDR = 0.054). (**f**) Phospho-ERK IHC staining in wildtype, *Foxp1-Shq1*$^{flox/flox}$, *Pten*$^{flox/flox}$, and *Foxp1-Shq1*$^{flox/flox}$;*Pten*$^{flox/flox}$ mouse prostate at 12 months. Scale bars, 50 µm. (**g**) Phospho-ERK IHC staining in *Foxp1-Shq1*$^{flox/flox}$;*Pten*$^{flox/flox}$ mouse prostate at 12 months showing the range of level over multiple fields. Scale bars, 50 µm.

(Supplementary Figure 5 - continued)

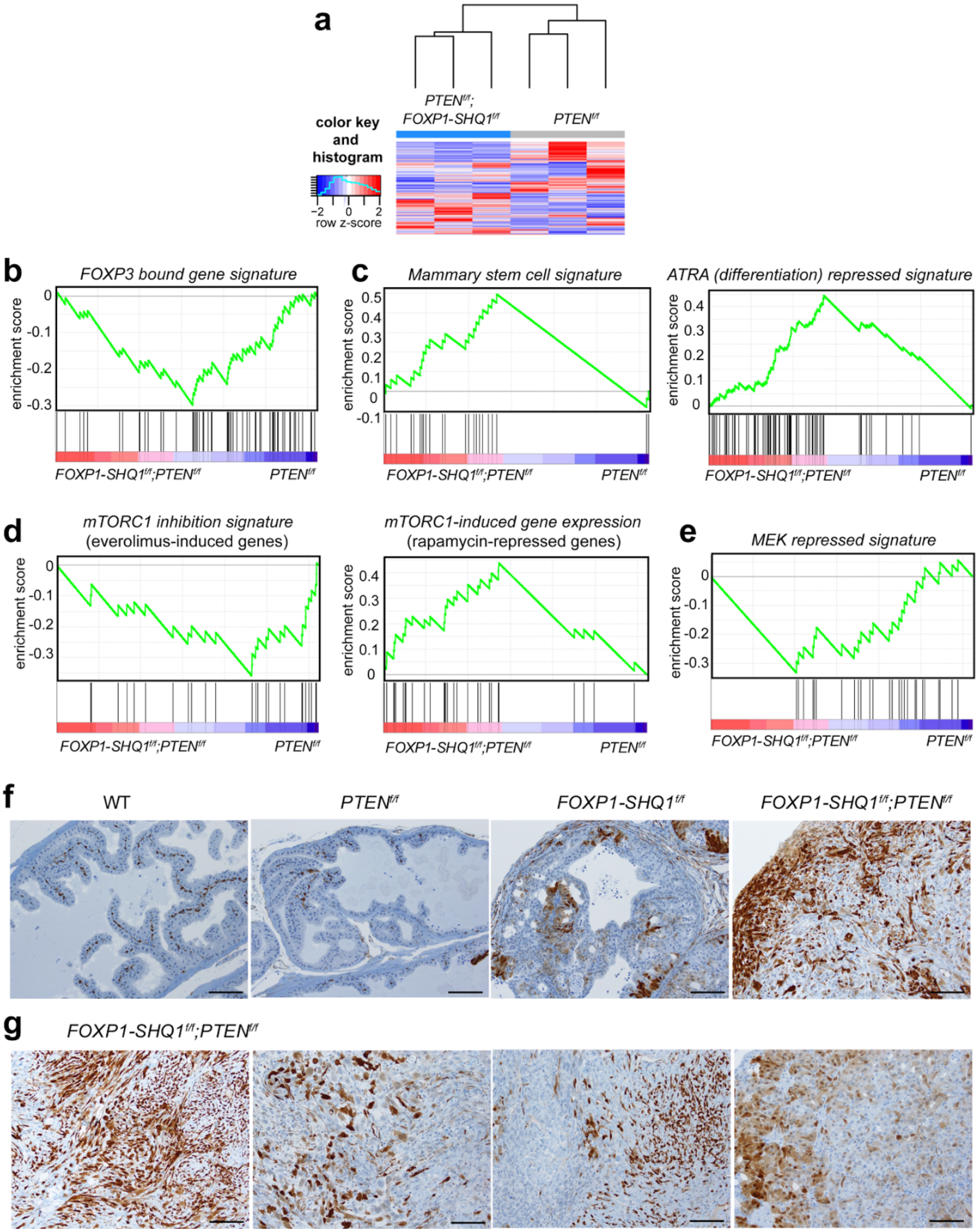

Supplementary Figure 6. Combined PTEN and SHQ1-FOXP1 locus loss correlates with restoration of AR responsive gene expression, despite unchanged AR RNA levels. (**a**) AR RNA levels (log_2 counts, RNAseq) in *Pten*$^{flox/flox}$ and *Foxp1-Shq1*$^{flox/flox}$*;Pten*$^{flox/flox}$ mouse prostate at 12 months (n=3 biological replicates each). (**b**) Heatmap of murine androgen-responsive (castration responsive) gene expression signature in *Pten*$^{flox/flox}$ and *Foxp1-Shq1*$^{flox/flox}$*;Pten*$^{flox/flox}$ mouse prostate at 12 months (n=3 each). As in Figure 3c but with gene names labeled and induction or repression by castration indicated. (**c**) Androgen-regulated signature score [5, 6] in primary human prostate adenocarcinoma (TCGA cohort) grouped by *FOXP1* loss (green) as a proxy for SHQ1-FOXP1 locus loss, *PTEN* loss (blue), neither (gray), or both (red), displayed by box plot. All pair-wise comparisons tested. t-test, *, P<0.05; **, P<0.01; ***, P < 0.001. (**d**) AR protein levels in *Pten*$^{flox/flox}$, *Foxp1-Shq1*$^{flox/flox}$, *Foxp1-Shq1*$^{flox/flox}$*;Pten*$^{flox/flox}$ mouse prostate at 12 months (n≥3 biological replicates each) by Western blot.

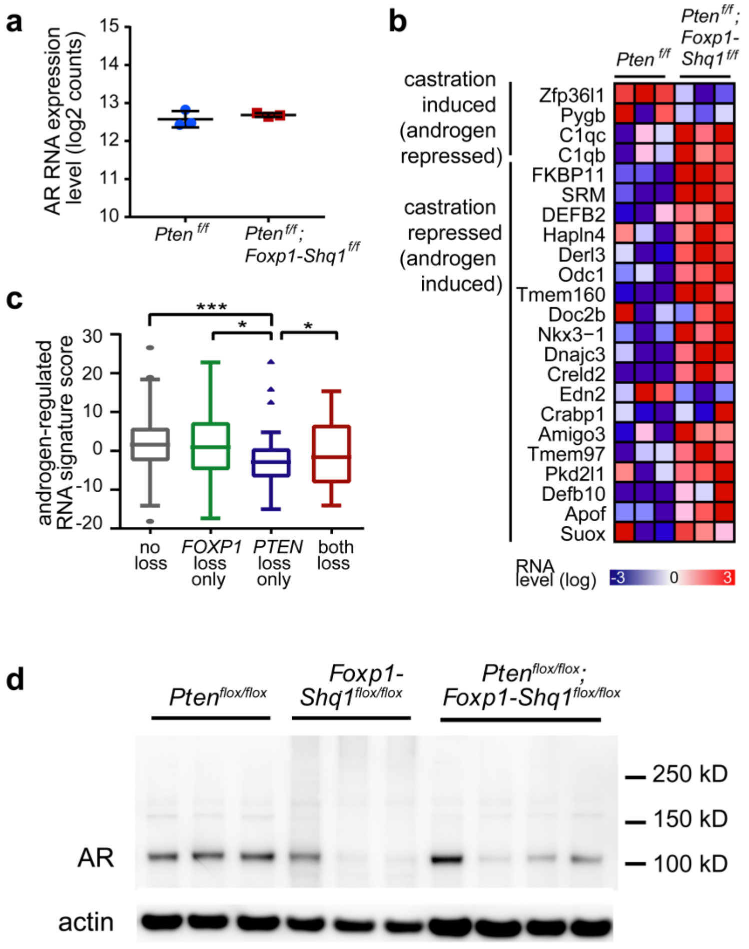

Supplementary Tables

Supplementary Table 1. Associations between *PTEN* copy number loss (hemi- or homozygous) and copy number loss of the *FOXP1-SHQ1* region and its constituent individual genes in primary prostate adenocarcinoma. The significance, P, of association by Fisher's Exact test is shown.

Cohort (primary non-neoadjuvant cases)	***Alteration 1***	***Alteration 2***	***P***
FOXP1-SHQ1 deletion			
MSKCC prostate adenocarcinoma (2010)[7]	*PTEN* loss	*FOXP1-SHQ1* loss (all genes)	0.008
Cornell/Broad neuroendocrine prostate cancer (2016)[8]	*PTEN* loss	*FOXP1-SHQ1* loss (all genes)	0.005
TCGA prostate adenocarcinoma (2015)[6]	*PI3CB* mutation (Y962N, E552K) or amplification alone	*FOXP1-SHQ1* loss (all genes)	0.002
Individual genes			
MSKCC prostate adenocarcinoma (2010)[7]	*PTEN* loss	*FOXP1* loss	0.001
MSKCC prostate adenocarcinoma (2010)[7]	*PTEN* loss	*SHQ1* loss	0.007
MSKCC prostate adenocarcinoma (2010)[7]	*PTEN* loss	*RYBP* loss	0.013
MSKCC prostate adenocarcinoma (2010)[7]	*PTEN* loss	*GPR27* loss	0.005
MSKCC prostate adenocarcinoma (2010)[7]	*PTEN* loss	*EIF4E3* loss	0.001
MSKCC prostate adenocarcinoma (2010)[7]	*PTEN* loss	*PROK2* loss	0.024
TCGA prostate adenocarcinoma (2015)[6]	*PTEN* loss	*FOXP1* loss	0.049
TCGA prostate adenocarcinoma (2015)[6]	*PTEN* loss	*SHQ1* loss	0.042
Cornell/Broad neuroendocrine prostate cancer (2016)[8]	*PTEN* loss	*FOXP1* loss	0.001
Cornell/Broad neuroendocrine prostate cancer (2016)[8]	*PTEN* loss	*SHQ1* loss	<0.001
Cornell/Broad neuroendocrine prostate cancer (2016)[8]	*PTEN* loss	*RYBP* loss	0.001
Cornell/Broad neuroendocrine prostate cancer (2016)[8]	*PTEN* loss	*GPR27* loss	0.004
Cornell/Broad neuroendocrine prostate cancer (2016)[8]	*PTEN* loss	*EIF4E3* loss	0.004
Cornell/Broad neuroendocrine prostate cancer (2016)[8]	*PTEN* loss	*PROK2* loss	0.004

Supplementary Table 2. Pan-cancer associations between *PTEN* copy number loss (hemi- or homozygous) and copy number loss of the *FOXP1-SHQ1* region and its constituent genes in TCGA cohorts. All published TCGA cohorts are shown. The significance, P, of association by Fisher's Exact test is shown.

Cohort	***Alteration 1***	***Alteration 2***	***P***
SHQ-FOXP1 deletion			
Breast Invasive Carcinoma (TCGA, 2015)[9]	*PTEN* loss	*FOXP1-SHQ1* loss (all genes)	<0.001
Stomach Adenocarcinoma (TCGA, 2014)[10]	*PTEN* loss	*FOXP1-SHQ1* loss (all genes)	0.001
Kidney Renal Clear Cell Carcinoma (TCGA, 2013)[11]	*PTEN* loss	*FOXP1-SHQ1* loss (all genes)	0.019
Lung Adenocarcinoma (TCGA, 2014)[12]	*PTEN* loss	*FOXP1-SHQ1* loss (all genes)	0.034
Lung Squamous Cell Carcinoma (TCGA, 2012)[13]	*PTEN* loss	*FOXP1-SHQ1* loss (all genes)	<0.001
Head and Neck Squamous Cell Carcinoma (TCGA, 2015)[14]	*PTEN* loss	*FOXP1-SHQ1* loss (all genes)	0.007
Uterine Corpus Endometrioid Carcinoma (TCGA, 2013)[15]	*PTEN* loss	*FOXP1-SHQ1* loss (all genes)	0.001
Bladder Urothelial Carcninoma (TCGA, 2014)[16]	*PIK3CA* amplification or mutation	*FOXP1-SHQ1* loss (all genes)	0.015
Individual genes			
Breast Invasive Carcinoma (TCGA, 2015)[9]	*PTEN* loss	*FOXP1* loss	<0.001
Breast Invasive Carcinoma (TCGA, 2015)[9]	*PTEN* loss	*EIF4E3* loss	<0.001
Breast Invasive Carcinoma (TCGA, 2015)[9]	*PTEN* loss	*GPR27* loss	<0.001
Breast Invasive Carcinoma (TCGA, 2015)[9]	*PTEN* loss	*PROK2* loss	<0.001
Breast Invasive Carcinoma (TCGA, 2015)[9]	*PTEN* loss	*RYBP* loss	<0.001
Breast Invasive Carcinoma (TCGA, 2015)[9]	*PTEN* loss	*SHQ1* loss	<0.001
Stomach Adenocarcinoma (TCGA, 2014)[10]	*PTEN* loss	*FOXP1* loss	<0.001
Stomach Adenocarcinoma (TCGA, 2014)[10]	*PTEN* loss	*EIF4E3* loss	<0.001
Stomach Adenocarcinoma (TCGA, 2014)[10]	*PTEN* loss	*GPR27* loss	<0.001
Stomach Adenocarcinoma (TCGA, 2014)[10]	*PTEN* loss	*SHQ1* loss	<0.001
Stomach Adenocarcinoma (TCGA, 2014)[10]	*PTEN* loss	*PROK2* loss	0.001
Stomach Adenocarcinoma (TCGA, 2014)[10]	*PTEN* loss	*RYBP* loss	0.004
Kidney Renal Clear Cell Carcinoma (TCGA, 2013)[11]	*PTEN* loss	*RYBP* loss	0.009
Kidney Renal Clear Cell Carcinoma (TCGA, 2013)[11]	*PTEN* loss	*SHQ1* loss	0.01
Kidney Renal Clear Cell Carcinoma (TCGA, 2013)[11]	*PTEN* loss	*EIF4E3* loss	0.019
Kidney Renal Clear Cell Carcinoma (TCGA, 2013)[11]	*PTEN* loss	*GPR27* loss	0.019
Kidney Renal Clear Cell Carcinoma (TCGA, 2013)[11]	*PTEN* loss	*PROK2* loss	0.019
Kidney Renal Clear Cell Carcinoma (TCGA, 2013)[11]	*PTEN* loss	*FOXP1* loss	0.021
Lung Adenocarcinoma (TCGA, 2014)[12]	*PTEN* loss	*RYBP* loss	0.028
Lung Adenocarcinoma (TCGA, 2014)[12]	*PTEN* loss	*FOXP1* loss	0.03
Lung Adenocarcinoma (TCGA, 2014)[12]	*PTEN* loss	*SHQ1* loss	0.034
Lung Adenocarcinoma (TCGA, 2014)[12]	*PTEN* loss	*EIF4E3* loss	0.041
Lung Adenocarcinoma (TCGA, 2014)[12]	*PTEN* loss	*GPR27* loss	0.041
Lung Adenocarcinoma (TCGA, 2014)[12]	*PTEN* loss	*PROK2* loss	0.041
Lung Squamous Cell Carcinoma (TCGA, 2012)[13]	*PTEN* loss	*FOXP1* loss	<0.001
Lung Squamous Cell Carcinoma (TCGA, 2012)[13]	*PTEN* loss	*EIF4E3* loss	<0.001
Lung Squamous Cell Carcinoma (TCGA, 2012)[13]	*PTEN* loss	*GPR27* loss	<0.001
Lung Squamous Cell Carcinoma (TCGA, 2012)[13]	*PTEN* loss	*PROK2* loss	<0.001
Lung Squamous Cell Carcinoma (TCGA, 2012)[13]	*PTEN* loss	*RYBP* loss	<0.001
Lung Squamous Cell Carcinoma (TCGA, 2012)[13]	*PTEN* loss	*SHQ1* loss	<0.001
Head and Neck Squamous Cell Carcinoma (TCGA, 2015)[14]	*PTEN* loss	*FOXP1* loss	<0.001
Head and Neck Squamous Cell Carcinoma (TCGA, 2015)[14]	*PTEN* loss	*EIF4E3* loss	<0.001

Head and Neck Squamous Cell Carcinoma (TCGA, 2015)[14]	*PTEN* loss	*GPR27* loss	<0.001
Head and Neck Squamous Cell Carcinoma (TCGA, 2015)[14]	*PTEN* loss	*PROK2* loss	<0.001
Head and Neck Squamous Cell Carcinoma (TCGA, 2015)[14]	*PTEN* loss	*RYBP* loss	0.001
Head and Neck Squamous Cell Carcinoma (TCGA, 2015)[14]	*PTEN* loss	*SHQ1* loss	0.001
Uterine Corpus Endometrioid Carcinoma (TCGA, 2013)[15]	*PTEN* loss	*FOXP1* loss	0.001
Uterine Corpus Endometrioid Carcinoma (TCGA, 2013)[15]	*PTEN* loss	*EIF4E3* loss	0.001
Uterine Corpus Endometrioid Carcinoma (TCGA, 2013)[15]	*PTEN* loss	*GPR27* loss	0.001
Uterine Corpus Endometrioid Carcinoma (TCGA, 2013)[15]	*PTEN* loss	*PROK2* loss	0.001
Uterine Corpus Endometrioid Carcinoma (TCGA, 2013)[15]	*PTEN* loss	*RYBP* loss	0.001
Uterine Corpus Endometrioid Carcinoma (TCGA, 2013)[15]	*PTEN* loss	*SHQ1* loss	0.001
Colorectal Adenocarcinoma (TCGA, 2012)[17]	*PTEN* loss	*FOXP1-SHQ1* loss, *FOXP1* loss, *EIF4E3* loss, *GPR27* loss, *PROK2* loss, *RYBP* loss, or *SHQ1* loss	NS
Kidney Chromophobe (TCGA, 2014)[18]	*PTEN* loss	*FOXP1-SHQ1* loss, *FOXP1* loss, *EIF4E3* loss, *GPR27* loss, *PROK2* loss, *RYBP* loss, or *SHQ1* loss	NS
Glioblastoma (TCGA, 2013)[19]	*PTEN* loss	*FOXP1-SHQ1* loss, *FOXP1* loss, *EIF4E3* loss, *GPR27* loss, *PROK2* loss, *RYBP* loss, or *SHQ1* loss	NS
Ovarian Serous Carcinoma (TCGA, 2011)[20]	*PTEN* loss	*FOXP1-SHQ1* loss, *FOXP1* loss, *EIF4E3* loss, *GPR27* loss, *PROK2* loss, *RYBP* loss, or *SHQ1* loss	NS

Supplementary Table 3. Combined *FOXP1* loss and *PTEN* loss is associated with recurrence in the MSKCC primary prostate cancer cohort. Multivariate Cox regression shows association of combined *FOXP1/PTEN* loss with biochemical recurrence in the MSK primary prostate cancer cohort [7]. HR, hazard ratio. CI, confidence interval.

				95% CI for HR	
	**B**	**P**	**HR**	**Lower**	**Upper**
**Indicator** (neither *FOXP1* nor *PTEN* loss)		.001			
***FOXP1* loss only** (expression z<-1 or hemi- or homozygous CN loss)	0.598	.226	1.818	.691	4.786
***PTEN* loss only** (expression z<-2 or hemi- or homozygous CN loss)	1.120	**.015**	**3.065**	**1.244**	**7.553**
**Both *FOXP1* and *PTEN* loss**	1.516	**.000**	**4.556**	**2.022**	**10.262**

Supplementary Methods

Transgenic mouse generation and genotyping

Foxp1-Shq1^{flox} conditional knockout transgenic mice were generated by targeting the *Shq1* conditional frameshift construct HTGRS0100_A_A12 (EUCOMM, KO first allele reporter-tagged insertion with conditional potential, diagram below, http://www.mousephenotype.org/data/alleles/MGI:1919421/tm1a(EUCOMM)Hmgu/) into *FOXP1*$^{f/f}$ ES cells generated from the *Foxp1*$^{f/f}$ conditional knockout mice described in Feng et al., 2010[21]. The targeting sequence with homology arms is delineated here https://www.i-dcc.org/imits/targ_rep/alleles/8745/targeting-vector-genbank-file. The vector was targeted into *FOXP1*$^{f/f}$ ES cells (agouti) and injected into C57BL/6 blastocysts by the Rockefeller University Gene Targeting Facility. After generation of the targeted transgenic mouse line, it was converted from a reporter-tagged knockout to a conditional knockout by breeding with a FLPase line (B6.Cg-Tg(ACTFLPe)9205Dym/J, Jackson Labs) and selection for loss of the neo cassette by PCR. Genotyping for *Shq1*-lox targeting was performed using the following primers: SHQ1-int3-F GTAGTGGCAGCTCACACTCGCCA and SHQ1-int3-R GAAGCAGGCCACCCAACTGACC (giving a single 527bp band for mice containing the unrecombined targeting construct). Genotyping for the *Shq1*-flox allele in the established transgenic line used primers CACCTGTGTTGCTAACGTTCCTTC and CTACTGTGGCTACTTCAAGATTACC. Genotyping the *Foxp1*-lox allele used the primers CACCCTCTCCAAGTCTGCCTCAG and CCAGGGATCAGAGATTACTGTAGC.

Foxp1-Shq1^{flox} mice were breed with *Pb-Cre4* mice (Jackson Labs strain 01XF5, PB-Cre4 B6.Cg-Tg(Pbsn-cre)4Prb/Nci) and *PTEN*$^{flox/flox}$ mice previously described.[22, 23] *PB-Cre4* is the *Cre* gene is driven by a composite promoter, $ARR_{2\ PB}$, which is a derivative of the rat *PB* promoter from which the *PB-Cre* was originally generated.[24] *PB-Cre4* mice express *Cre* at high levels and at high penetrance. All animal work was done under IACUC approved guidelines.

SHQ1-flox targeting construct HTGRS0100_A_A12:

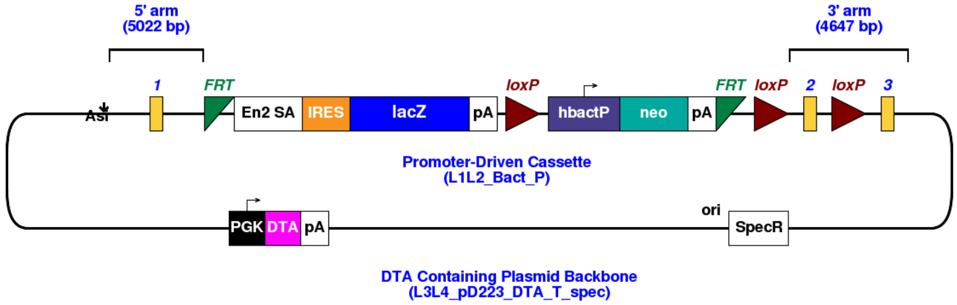

Genomic PCR and RT-PCR from mouse tumors

For genomic DNA PCR, DNA was extracted from FFPE slides using the AllPrep DNA/RNA FFPE Kit (Qiagen). Primers amplifying the floxed or recombined allele of *Foxp1* (AGAAGATCCGTTGACCTGCA, GAACACTGTCGAATGACCCTGC, and

ACGTGCCCATTTCTTCAGGT), wild-type, floxed, or recombined *Shq1* (CACCTGTGTTGCTAACGTTCCTTC, CTACTGTGGCTACTTCAAGATTACC, and GGGTTTCTTCACTGCTCGAG), and the recombined allele of the entire *Foxp1-Shq1* locus (AGAAGATCCGTTGACCTGCA, GCATAACGATACCACGATATCAA) were used for 38 cycles of PCR. The PCR products were resolved on 1.5% agarose gels and visualized using ethidium bromide staining.

For quantitative RT-PCR, RNA isolated from frozen tissue (1.5µg) was converted to cDNA using the High-Capacity RNA-to-cDNA kit (ThermoFisher), and analyzed by real-time PCR using 2x QuantiFast SYBR Green PCR Master Mix (Qiagen). Primers specific for the full-length unrecombined alleles of *Foxp1* (AGAACGCGGAAGTTAGACCA and GCTGCTTTTCTGGAGATTC) and *Shq1* (GTACTTCGAGGGGGTGGACT and CATAGGTCCCCTGCTCAGAT) were used, and the Ct values were normalized to *GAPDH* (TGCACCACCAACTGCTTAGC and GGCATGGACTGTGGTCATGAG).

References

1. Zheng Y, Josefowicz SZ, Kas A, Chu TT, Gavin MA, Rudensky AY. Genome-wide analysis of Foxp3 target genes in developing and mature regulatory T cells. *Nature* **445**, 936-940 (2007).

2. Pece S*, et al.* Biological and molecular heterogeneity of breast cancers correlates with their cancer stem cell content. *Cell* **140**, 62-73 (2010).

3. Majumder PK*, et al.* mTOR inhibition reverses Akt-dependent prostate intraepithelial neoplasia through regulation of apoptotic and HIF-1-dependent pathways. *Nature medicine* **10**, 594-601 (2004).

4. Creighton CJ, Hilger AM, Murthy S, Rae JM, Chinnaiyan AM, El-Ashry D. Activation of mitogen-activated protein kinase in estrogen receptor alpha-positive breast cancer cells in vitro induces an in vivo molecular phenotype of estrogen receptor alpha-negative human breast tumors. *Cancer research* **66**, 3903-3911 (2006).

5. Hieronymus H*, et al.* Gene expression signature-based chemical genomic prediction identifies a novel class of HSP90 pathway modulators. *Cancer cell* **10**, 321-330 (2006).

6. Cancer Genome Atlas Research N. The Molecular Taxonomy of Primary Prostate Cancer. *Cell* **163**, 1011-1025 (2015).

7. Taylor BS*, et al.* Integrative genomic profiling of human prostate cancer. *Cancer cell* **18**, 11-22 (2010).

8. Beltran H*, et al.* Divergent clonal evolution of castration-resistant neuroendocrine prostate cancer. *Nature medicine* **22**, 298-305 (2016).

9. Ciriello G*, et al.* Comprehensive Molecular Portraits of Invasive Lobular Breast Cancer. *Cell* **163**, 506-519 (2015).

10. Cancer Genome Atlas Research N. Comprehensive molecular characterization of gastric adenocarcinoma. *Nature* **513**, 202-209 (2014).

11. Cancer Genome Atlas Research N. Comprehensive molecular characterization of clear cell renal cell carcinoma. *Nature* **499**, 43-49 (2013).

12. Cancer Genome Atlas Research N. Comprehensive molecular profiling of lung adenocarcinoma. *Nature* **511**, 543-550 (2014).

13. Cancer Genome Atlas Research N. Comprehensive genomic characterization of squamous cell lung cancers. *Nature* **489**, 519-525 (2012).

14. Cancer Genome Atlas N. Comprehensive genomic characterization of head and neck squamous cell carcinomas. *Nature* **517**, 576-582 (2015).

15. Cancer Genome Atlas Research N*, et al.* Integrated genomic characterization of endometrial carcinoma. *Nature* **497**, 67-73 (2013).

16. Cancer Genome Atlas Research N. Comprehensive molecular characterization of urothelial bladder carcinoma. *Nature* **507**, 315-322 (2014).

17. Cancer Genome Atlas N. Comprehensive molecular characterization of human colon and rectal cancer. *Nature* **487**, 330-337 (2012).

18. Davis CF*, et al.* The somatic genomic landscape of chromophobe renal cell carcinoma. *Cancer cell* **26**, 319-330 (2014).

19. Brennan CW*, et al.* The somatic genomic landscape of glioblastoma. *Cell* **155**, 462-477 (2013).

20. Cancer Genome Atlas Research N. Integrated genomic analyses of ovarian carcinoma. *Nature* **474**, 609-615 (2011).

21. Feng X*, et al.* Foxp1 is an essential transcriptional regulator for the generation of quiescent naive T cells during thymocyte development. *Blood* **115**, 510-518 (2010).

22. Carver BS*, et al.* Reciprocal feedback regulation of PI3K and androgen receptor signaling in PTEN-deficient prostate cancer. *Cancer cell* **19**, 575-586 (2011).

23. King JC*, et al.* Cooperativity of TMPRSS2-ERG with PI3-kinase pathway activation in prostate oncogenesis. *Nature genetics* **41**, 524-526 (2009).

24. Wu X*, et al.* Generation of a prostate epithelial cell-specific Cre transgenic mouse model for tissue-specific gene ablation. *Mechanisms of development* **101**, 61-69 (2001).
